# Dengue in Europe

**Published:** 1890-03-08

**Authors:** 


### The Practitioner's Mirror and Retrospect.

[*The Editor will be glad to receive offers of co-operation and contributions from members of the profession. There is no doubt that much valuable material full of interest and instruction is at present lost to science. This is largely so in the case of* (1) *the younger and less-known members of the hospital staff* (2) *those connected with cottage hospitals, and* (3) *the great body of medical practitioners not attached to any institution. The co-operation of all is cordially invited to enable the Editor to make* The Hospital *of the utmost possible use and value to the profession. Specialists willing to review books on their special subjects are invited to communicate this fact at once. All letters should be addressed to* The Editor, The Lodge, Porchester Square, London, W.]

### MEDICINE.

### DENGUE IN EUROPE.

There can scarcely be a doubt but that some relation subsists between the two epidemic diseases known as dengue and influenza, though opinions are divided as to whether they should be regarded as varieties of the same disease dependent on climatic and other conditions, or as distinct, though allied, species, as it were, of one genus. Influenza has been observed in every climate, though apparently more prevalent in temperate regions and the winter months, while dengue had until recently scarcely extended itself beyond the tropics.

This acclimatisation, and partial naturalisation, so to say, of a disease is not altogether a new phenomenon, cholera and yellow fever presenting good illustrations both of epidemic waves and of steady and permanent progress; while it would now appear that dengue must be added to those which are surely though slowly extending their geographical areas.

Not wholly unknown even on the Mediterranean to our ancestors, it seems to have broken out with unprecedented severity in India in 1824, and during the following years to have invaded the West Indies and the mainland of both the northern and southern Continents enclosing the Gulf of Mexico. Twenty-three years later it re-appeared there as an epidemic, and after a like interval, in 1870, when it spread all over India and East Africa. There have been minor epidemics in India, but of more immediate interest to us is its gradual establishment in the Levant. It is just 45 years since it took up its home in Egypt, spreading in ten years over the whole of Northern Africa. Between 1855 and last year its progress was slow, creeping along the shores of Syria and the island of Cyprus. But in 1889 it burst out with fresh vigour, invading not only the seaboard but the entire area of Syria and Asia minor, as well as the northern and western shores of the Archipelago, and all the islands except Crete. As yet the Morea and Adriatic have escaped, but there seems every probability that, whether after an interval of quiescence or not, it will ere long invade Italy and the south of Europe.

The epidemic of last year appears to have broken out simultaneously at Beyrout and other places in June. In Beyrout by the end of August 20,000 persons in a summer population of 100,000 had been attacked; in Smyrna 150,000 out of 200,000; and at Jaffa nearly the entire population. In the Lebanon it did not spare villages even at elevations of 4,000 to 5,000 feet above the sea level. It advanced with extraordinary rapidity, Smyrna and Constantinople being reached within a few days of one another, and a few weeks later Salonika, and even Athens.

Though new to Asia Minor, and at first causing some embarrassment to the native medical practitioners, it was at once recognised by all who had resided in Egypt or Syria as dengue, or the *Abou-rekab* of the Arabs. Dr. De Brun, of Beyrout, from numerous observations, some amounting to crucial experiments, fixes the incubation period at about 36 hours, in which, as in its now undoubted communicability, it resembles influenza. Though certainly itself a specific disease, it combines many of the symptoms of scarlatina, rheumatism, and influenza, and is remarkable for exhibiting, at least in many instances, two distinct exanthems, an initial and a consecutive, the former being often absent.

Dr. Leoni describes the symptoms as sudden access of fever, often, but not always, preceded by rigors, the temperature rising in a few hours to 40 deg. or 41 deg. C. (104 deg. or 105.8 deg. F.), and invariably attended by severe pains in the loins, and more or less generally elsewhere, especially in the lower limbs. There is agonising headache, vertigo, nausea, or vomiting, with a sense of oppression at the heart, and extreme prostration. The urine is scanty, but not albuminous or bloody, and the spleen is not enlarged. There are no physical signs in the lungs, nor is the mind affected as a rule.

The pyrexia lasts twenty-four, forty-eight, or even seventy-two hours, when it begins to abate, sometimes falling rapidly, at other times slowly over four or five days. The defervescence is accompanied by profuse perspiration and an eruption resembling that of rubeola, but as irritable as uriticaria, which may appear even before the decline of the fever. Recovery is tedious, the temperature remaining slightly raised, the tongue furred, the appetite wanting, and the limbs weak for some time.

The mortality is, however, almost insignificant viewed as a percentage, but the symptoms may be so aggravated that the patient sinks from sheer asthenia, from irrepressible vomiting, from epistaxis, or, more rarely, from the intensity of the pyrexial and cerebral symptoms, delirium, coma, and convulsions.

Dr. De Brun mentions a remarkable case in which an attack of dengue cured a long standing facial neuralgia that had resisted every imaginable treatment, and was so severe that the patient had frequently passed a whole day without taking any nourishment on account of the excruciating pain involved in the least movement of the jaws.

Antipyrin and saline aperients, with quinine, &c., during convalescence, seem to be the best treatment for dengue as for influenza, but though the attack itself is more painful and intense, the danger arising from pulmonary complications incident to influenza is absent in this disease, which, too, seems more prevalent in the warmer months of the year.

